# Non-Aβ-Dependent Factors Associated with Global Cognitive and Physical Function in Alzheimer’s Disease: A Pilot Multivariate Analysis

**DOI:** 10.3390/jcm8020224

**Published:** 2019-02-09

**Authors:** Anna Pedrinolla, Massimo Venturelli, Stefano Tamburin, Cristina Fonte, Anna Maria Stabile, Ilaria Boscolo Galazzo, Barbara Ghinassi, Mary Anna Venneri, Francesca Benedetta Pizzini, Ettore Muti, Nicola Smania, Angela Di Baldassarre, Fabio Naro, Mario Rende, Federico Schena

**Affiliations:** 1 Departement of Neuroscience, Biomedicine and Movement Sciences, University of Verona, Via Casorati 43, 37127 Verona, Italy; anna.pedrinolla@univr.it (A.P.); stefano.tamburin@univr.it (S.T.); cristina.fonte@univr.it (C.F.); nicola.smania@univr.it (N.S.); federico.schena@univr.it (F.S.); 2 Department of Internal Medicine, University of Utah, Salt Lake City, UT 84132, USA; 3 Neuromotor and Cognitive Rehabilitation Research Centre, University of Verona, 37134 Verona, Italy; 4 Department of Surgical and Biomedical Sciences, Section of Human Anatomy, School of Medicine, University of Perugia, 06123 Perugia, Italy; anna.stabile@unipg.it (A.M.S.); mario.rende@unipg.it (M.R.); 5 Department of Computer Science, University of Verona, 37134 Verona, Italy; ilaria.boscologalazzo@univr.it; 6 Department of Medicine and Aging Sciences, University G. d’Annunzio, Chieti-Pescara, 66100 Chieti, Italy; barbara.ghinassi@unich.it (B.G.); dibaldas@unich.it (A.D.B.); 7 Department of Anatomical, Histological, Forensic Medicine and Orthopedic Science, 00185 Rome, Italy; maryanna.vennere@uniroma1.it (M.A.V.); fabio.naro@uniroma1.it (F.N.); 8 Neuroradiology, Department of Diagnostics and Pathology, Verona University Hospital, 37134 Verona, Italy; francesca.pizzini@univr.it; 9 Mons. Mazzali Foundation, 46100 Mantua, Italy; ettoremuti@fondazionemazzali.it

**Keywords:** Alzheimer’s disease, mild cognitive impairment, dementia, exercise, memory, physical activity

## Abstract

Recent literature highlights the importance of identifying factors associated with mild cognitive impairment (MCI) and Alzheimer’s Disease (AD). Actual validated biomarkers include neuroimaging and cerebrospinal fluid assessments; however, we investigated non-Aβ-dependent factors associated with dementia in 12 MCI and 30 AD patients. Patients were assessed for global cognitive function (Mini-Mental state examination—MMSE), physical function (Physical Performance Test—PPT), exercise capacity (6-min walking test—6MWT), maximal oxygen uptake (VO_2_max), brain volume, vascular function (flow-mediated dilation—FMD), inflammatory status (tumor necrosis factor—α ,TNF- α, interleukin-6, -10 and -15) and neurotrophin receptors (p75NTR and Tropomyosin receptor kinase A -TrkA). Baseline multifactorial information was submitted to two separate backward stepwise regression analyses to identify the variables associated with cognitive and physical decline in demented patients. A multivariate regression was then applied to verify the stepwise regression. The results indicated that the combination of 6MWT and VO_2_max was associated with both global cognitive and physical function (MMSE = 11.384 + (0.00599 × 6MWT) − (0.235 × VO_2_max)); (PPT = 1.848 + (0.0264 × 6MWT) + (19.693 × VO_2_max)). These results may offer important information that might help to identify specific targets for therapeutic strategies (NIH Clinical trial identification number NCT03034746).

## 1. Introduction

Dementia is a common age-related disorder leading to cognitive and functional impairments [1,2], affecting the quality of life of patients [3]. The global prevalence is estimated to be as high as 24 million and is expected to increase as people live longer, doubling every 20 years, predicted to reach 81.1 million worldwide by 2040 [1]. Older individuals can have demonstrable cognitive impairment without crossing the threshold for dementia and this condition has been termed “mild cognitive impairment” (MCI) [2]. MCI is characterized by cognitive changes that are greater than expected for an individual’s age and education level but do not severely interfere with daily-life activities [3]. Considerably important is the fact that individuals with MCI have an increased risk of developing Alzheimer’s disease (AD) [2,4]. AD is the most common form of dementia, characterized by a progressive deterioration of cognitive function due to widespread neuronal loss and synaptic dysfunction, accompanied by extracellular amyloidosis and intracellular tangle formation [5]. Current literature suggests that patients with MCI progress to AD at a rate of 10% to 15% per year, and 80% of these patients converted to AD after approximately 6 years of follow-up [2]. 

In recent decades, much effort has been dedicated to the early clinical diagnosis of dementing disorders with the aim of identifying factors associated with AD [6]. Most of the studies have mainly focused on the association of cognitive decline with biomarkers obtained by neuroimaging and from cerebrospinal fluid [4,5,7,8]. However, it has been demonstrated that cognitive deterioration, accumulation of amyloid-β (Aβ) and tau protein are not equivalent and not linear with respect to disease progression and the appearance of its cognitive symptoms [9].

Interestingly, other recent studies provided evidence about the polyhedral etiology of AD [10,11,12,13,14], demonstrating that AD symptoms and dysfunctions are not confined to the cognitive and executive level, but range over several physical and bioenergetic functions [15,16,17,18,19]. Moreover, it has been demonstrated that cardiorespiratory fitness, which is closely related to the level of physical activity, is strongly related with the rate of cognitive decline [20,21]. These recent findings may indicate that some non-Aβ-dependent factors may be associated with AD. Importantly, recognizing variables that can be associated with MCI and AD and can be easily evaluated within the clinical setting is of primary importance. Moreover, the early recognition of cognitive decline would promote the appropriate implementation of preventive approaches. 

In light of this premise, the aim of this study was to explore the association, with a multivariate approach, between global cognitive function, physical function and a wide panel of non-Aβ-dependent factors potentially associated with MCI and AD. Our working hypothesis was that, due to the several physiological mechanisms involved in the AD pathogenesis of the disease, non-Aβ-dependent variables (i.e., metabolic, inflammatory, vascular, and functional biomarkers) would transpire to be associated with this neurodegenerative disease.

## 2. Methods

*Participants*. Patients with MCI with positive biomarkers for AD and patients with AD were recruited from the Neuromotor and Cognitive Rehabilitation Research Center of Azienda Ospedaliera Universitaria Integrata of Verona, and the Geriatric Institute Mons. Arrigo Mazzali Foundation (Mantua, Italy). Clinical diagnosis of MCI with biomarkers for AD and probable AD was established according to the National Institute on Aging-Alzheimer’s Association diagnostic guideline for MCI due to AD and AD [19,22,23]. Patients with MCI with probable biomarkers for AD and patients with AD were screened, including health history, physical examination, blood pressure assessment, blood sample, level of physical activity, and familiarization with the study procedures. Other exclusion criteria were as follows: history of depression or psychosis, alcohol or drug abuse, other neurological disorders (e.g., Parkinson’s disease, traumatic brain injury, stroke, multiple sclerosis), cardiac, orthopedic (e.g., osteoarthrosis) or respiratory (e.g., chronic obstructive pulmonary disease) conditions. All procedures were conducted after informed and written consent was obtained from the patients and their relatives in accordance with the Declaration of Helsinki, as part of a protocol approved by the Institutional Review Board of the Azienda Ospedaliera Universitaria Integrata (Verona, Italy—#2389; NIH Clinical trial identification number: NCT03034746). 

*Assessment procedure and study overview.* Neurologists and clinical neuropsychologists with specific expertise in dementia investigated the cognitive profile of the patients with a full neuropsychological profile and the following tests were performed: Mini-Mental State Examination (MMSE) was used to assess the global cognitive status; Clinical Dementia Rating (CDR) scale was administered to quantify the severity of dementia; the Italian version of the Frontal Assessment Battery (FAB) was used to assess executive functions. Kinesiologist performed physical tests. The Physical Performance Test (PPT) was used to assess physical function competences, while the test for the estimate of maximal oxygen consumption (VO_2_max) and 6-min walking test (6MWT) were used to assess bioenergetic and endurance aspects. The flow-mediated dilation (FMD) test, performed by an expert and skilled radiologist, was used for assessing the vascular function, while an MRI examination was performed to derive brain volume measures. Patients with MCI and AD included in this study underwent three testing sessions on three different days. On the first day, participants were evaluated for cognitive profile and underwent a blood sample. On the second day, participants performed PPT, 6MWT, and VO_2_max tests, while on the third day, participants underwent an FMD test and MRI. 

*Level of physical activity*. The International Physical Activity Questionnaire (IPAQ) was used to estimate the level of physical activity of the participants. Each question was administered to patients and their caregivers.

*Blood sample and analysis.* Venous peripheral blood (25 mL) was collected between 9:00 and 10:00 am in a fasted state and processed within 2 h to separate monocytes (Mono). From a different vacutainer, plasma was separated from peripheral blood by centrifugation (1200 rpm for 20 min at 4 °C) and kept at −80 °C until analysis. By Fluorescence Activated Cell Scanning (FACS) Scan analysis, Tropomyosin receptor kinase A -TrKA and p75 neurotrophins -p75NTR- receptor expression (expressed as % of positive cells and Mean Fluorescent Intensity: MFI) and interleukin (IL) 6 and 10 were evaluated on monocytes. More details of the methods are given in our previous paper. Plasmatic NGF concentration was measured by enzyme immunoassay ELISA (DuoSet ELISA development R&D System, #DY256-05) according to the manufacturer’s protocol [24]. Plasmatic VEGF and TNFα concentrations were measured by enzyme immunoassay ELISA (DuoSet ELISA development R&D System, VEGF Human ELISA Kit #DY256-0 Invitrogen # KHG0111 and TNF alpha Human ELISA Kit Invitrogen #BMS223-4) [24]. The plasmatic concentration of IL-15 was measured by enzyme immunoassay ELISA (RAYBIOTECH) according to the manufacturer’s protocol [24]. We decided to perform some measures on peripheral monocytes and some on plasma since the former are recognized to have a pivotal role in the pathogenesis of AD, while the latter is a non-neurological sample that is useful for the evaluation of peripheral biomarkers of AD [24]. 

*Physical performance test (PPT)*. The nine-item PPT assesses physical function competences. The following maneuvers simulating daily activities were assessed: writing a sentence; simulation of eating; rising up and putting a heavy book on a shelf; dressing and taking off a jacket; picking up a coin from the floor; turning 360°; gait test; climbing stairs; number of stairs climbed. Seven of the nine tasks were timed and the time interval scores of each task were given, from 0 if the task was unable to be performed to 4 if it was performed as well as it could be. During the 360° turn, stability and continuity of turning were assessed. The maximum score for the nine items was 36 points [25]. 

*Maximal oxygen consumption (VO_2_max).* Before performing the VO_2_max test, participants were instructed to find the self-selected speed (SSS, the most comfortable speed) on the treadmill. The test started from 0.5 km/h and the speed was increased by 0.1 km/h every 30 s until the patients referred to walk comfortably [26]. Subsequently, after a 15-min break, subjects performed a 3-speed walking test on a treadmill (Run Race, Technogym, Gambettola, Italy). A portable metabolimeter (K4b^2^, Cosmed, Rome, Italy) was used for cardiopulmonary measures. The test consisted of 2 phases: in the first phase, the subjects were asked to stand in the resting condition for 2 min while the resting oxygen uptake and heart rate (HR) were recorded. The second phase consisted of three 5-min bouts of walking at 80%, 100% and 120% of SSS. The initial minutes of walking secured a steady state VO_2_. For any bout, the average of the last minute VO_2_ measurements, and HR were calculated. After having defined the HR/VO_2_ relation using the linear regression, VO_2_max was than calculated by means of the following formula:

$${\mathrm{VO}}_{2}\max=(\mathrm{HRmax}-q)/m$$

where HRmax = theoretical HR max calculated using Karvonen formula (220-age); q = intercepts; m = angular coefficient. 

*Six-minute walking test (6MWT).* The 6MWT measures the maximum distance that a person can walk over 6 min and it is commonly used as an assessment of exercise capacity. The participants were instructed to walk from one end of a 15-meter course to the other and back again as many times as possible in 6 min, while under the supervision of a kinesiologist. After each minute, participants were informed of the time elapsed and were given standardized encouragement. The distance (meters) covered in 6 min was recorded [27]. 

*Flow-mediated dilation (FMD).* The FMD test was performed in a quiet room with participants in abstinence from alcohol, antioxidants (i.e., orange juice, beetroot juice) and caffeine for at least 12 h [28]. High-resolution ultrasound was used to image the brachial artery at rest and after 5 min of ischemia. All the FMD tests were performed with the participant in the supine position, with the right arm extended at an angle of ~90° from the torso. The brachial artery was imaged using a high-resolution ultrasound system Logiq-7 ultrasound Doppler system (General Electric Medical Systems, Milwaukee, WI, USA). The ultrasound Doppler system was equipped with a 12–14 MHz linear array transducer. The brachial artery was imaged 5–10 cm above the antecubital fossa in the longitudinal plan, and the diameter was determined at 90° angle along the central axis of the scanned area. When an optimal image was acquired, the position was maintained for the whole test and all scans were stored for later analyses. After baseline brachial artery imaging, a blood pressure cuff was placed around the forearm and inflated to 250 mmHg for 5 min. Brachial artery images and blood velocity were obtained continuously 30 s before and 2 min after cuff release [28]. The brachial artery images were analyzed by a blinded investigator by means of FloWave.US [29]. Arterial diameter was measured as the distance (mm) between the intima-lumen interfaces for the anterior and posteriors walls. Utilizing arterial diameter and the blood velocity, blood flow (BF) was calculated as follows:

BF (mL/min) = blood velocity · ᴨ · (vessel diameter/2)^2^ · 60

The calculation of FMD as a percentage change uses the peak diameter in response to reactive hyperemia in relation to the baseline diameter and was calculated using the following equation:

FMD (%) = (peak diameter-baseline diameter)/baseline diameter

and when multiplied by 100, FMD is expressed as a percentage of change in the vessel caliber [28]. 

The post cuff release shear rate was calculated using the following equation: Shear rate (s^−1^) = 8 V_mean_/vessel diameter. The cumulative shear rate (s^−1^ · s) and the reactive post cuff release (total blood flow over 2 min) were integrated using the trapezoidal rule and calculated as:

∑[yi(x(i+1)−xi)+(12)(y(i+1)−yi)(x(i+1)−xi)]

Consequently, FMD was normalized to the shear rate (FMD/Shear Rate) [28].

*Brain volume.* All patients were scanned on a 3T Philips Achieva MRI system equipped with an 8-channel head coil. Subjects were instructed to remain as still as possible during scanning to reduce motion artefacts in the collected images. A 3D T1-weighted turbo field echo anatomical scan was acquired in all subjects (TR/TE = 8.16/3.73 ms; 180 slices, 1 × 1 × 1 mm^3^). Brain tissue volume, both non-normalised and normalised for subject head size, was estimated with SIENAX [30,31], as included in FSL 5.0.9. SIENAX starts by extracting brain and skull images from the single whole-head input data. The brain image is then affine-registered to the MNI152 space template and tissue-type segmentation with partial volume estimation is carried out in order to calculate the total volume of brain tissue (TBV). This parameter of interest was obtained as the sum of measured cortical and subcortical gray matter (GM) and white matter (WM) volumes, including the brainstem and cerebellum [32].

*Statistical analysis.* Data are presented as mean ± SD. First, normality was assessed by the Shapiro–Wilk test. The Pearson correlation coefficients between both MMSE/PPT and all the measured variables were calculated. Predictive analyses were performed considering the variables correlated with MMSE and PPT (P-to enter = 0.1). Two separated backward stepwise regression analyses were conducted for the selected variables to identify the most predictable variables. Finally, a multivariate regression (enter method) was used to verify the stepwise regression analyses and obtain the final equation. In the final resultant model, the significance was set at *p* = 0.05. All statistical analyses were performed with SigmaPLOT Windows Version 14.0 (Systat Software, Chicago, IL, USA).

## 3. Results

*Subjects’ characteristics.* The demographic and clinical characteristics of the study participants are displayed in Table 1. Forty-two individuals (MCI with positive biomarkers for AD:12, AD:30) were included in this study. Drugs for AD and other medications taken by the patients with MCI and AD are also displayed in Table 1. The data concerning all the assessed variables are displayed in Table 2. 

*Correlations between global cognitive function, physical function and non-Aβ-dependent variables*. The analysis of correlations by means of the Pearson correlation coefficient showed significant correlations between MMSE and IADL (*p* < 0.001), 6MWT (*p* < 0.001), VO_2_max (*p* < 0.001), β-NGF (*p* = 0.004), HCT (*p* = 0.015), TrkA Mono (*p* = 0.020), and MFIp75NTR (*p* = 0.020). The same analysis revealed significant correlations between PPT and IADL (*p* < 0.001), 6MWT (*p* < 0.001), VO_2_max (*p* = 0.004), β-NGF (*p* = 0.015), MFIp75NTR (*p* < 0.001), and IL-10 (*p* = 0.016) (Table 3). 

*Multivariate approach*. Table 4 and Table 5 display the results of the two separate backward stepwise regression analyses followed by a multivariate regression conducted to determine the linear relationship between non-Aβ-dependent variables and MMSE and PPT. The final result showed that only 6MWT (*p* = 0.029) and VO_2_max (*p* = 0.087) were kept in the regression by the analysis for the prediction of MMSE (Table 4) and PPT, 6MWT (*p* < 0.001) and VO_2_max (*p* = 0.079) (Table 5). 

## 4. Discussion

Although the early recognition of biomarkers potentially associated with MCI and AD is a major target of clinical and pre-clinical research, not a single assessment-battery has been adopted as standard clinical care. In the present study, we explored the association between global cognitive function, physical function and several non-Aβ-dependent factors potentially associated with MCI and AD, easily quantifiable within several clinical settings. In accordance with our hypothesis, global cognitive function and physical function, measured by MMSE and PPT, were associated with 6MWT and VO_2_max. It is important to note that although VO_2_max was kept in the two final models, it was only marginally significant. Importantly, the 6MWT was the factor most related with both global cognitive function and physical function in MCI and AD.

*Global cognitive function and physical function and their predictors.* The statistical approach used to identify the relation between the measured variables and the global cognitive function in demented individuals highlighted a positive relation between exercise capacity and aerobic capacity (Table 4). This result suggests that dementia is a process which includes other pathways beyond Aβ deposition. In previous studies, exercise capacity has been shown to be strictly associated with gray matter volumes, as well as with memory and processing function [27,33]. Makizako and colleagues [27] recruited 91 community-dwelling older adults with MCI who completed a 6MWT and underwent a structural MRI scanning, as well as memory tests. With this study, the authors provided evidence about the positive relation between exercise capacity and memory function, and both variables were related to the gray matter volume. Furthermore, Welmer and colleagues [33] provided further evidence about the relation between walking speed and its association with processing speed and executive function in demented and non-demented elderly individuals. In demented people, cognitive dysfunction is often accompanied by a reduction of functional performances [17,30,34]. Moreover, other recent studies have highlighted the correlation between brain integrity and aerobic capacity in non-demented and demented people. Burns and colleagues [20] demonstrated that aerobic capacity was strictly related with brain atrophy in the earliest clinical stage of AD. In that study, the authors showed that a higher fitness level in early stage AD participants was associated with preserved brain volume independent of age and dementia severity. Perea and colleagues [21] also demonstrated that aerobic capacity is associated with increased white matter integrity in the early stage of AD, supporting our results.

Consequently, we explored the relationship between non-Aβ-dependent variables and physical function to find variables related with functional performance. Like MMSE, the result of our multivariate approach generated two variables, specifically, 6MWT and VO_2_max (Table 5). Although some studies have provided evidence about the correlation between 6MWT and aerobic capacity with the severity of dementia, to our knowledge no studies have investigated the relation between those variables and PPT in MCI and AD. Bronas and colleagues [31] investigated the association between aerobic capacity tested by means of a direct test, the 6MWT, and a physical performance test in individuals with AD. The authors identified a significant correlation between all these variables, indicating that the 6MWT was related to physical performance in this clinical population, supporting the results of our study. 

*Exercise as a potential strategy for counteracting the development of dementia.* Interestingly, data from the current study suggest that the variable most associated with cognitive and functional impairment was exercise capacity, in this context measured by the 6MWT. Greater exercise capacity reflects higher levels of physical activity as well as a higher aerobic fitness level [27]. As we mentioned previously, all of these parameters have been associated with maintenance of cognitive function and decreased risk of developing dementia [27,35], suggesting that maintaining or increasing levels of physical activity might be a useful strategy to counteract the deleterious effects of this neurodegenerative disease [36,37,38]. Boots and colleagues [39] recently reported that a higher exercise capacity level is associated with better brain health and cognition, as well as with the preservation of critical brain areas, reduced brain atrophy, and preserved white matter microstructure in several populations including both cognitively healthy older adults and demented individuals. The same evidence has been previously reported by other authors. Bronas and colleagues [31] provided evidence that exercise capacity is associated with improved or maintained brain structure and cognitive function; Loprinzi and colleagues [40] reported that maintaining a good exercise capacity helps in maintaining cognitive and memory functions in demented individuals. More in detail, Makizako and colleagues [27] demonstrated that higher exercise capacity in individuals with MCI is accompanied by greater brain volume in specific brain areas such as left middle temporal gyrus, middle occipital gyrus, and hippocampus. Interestingly, the role of exercise capacity in brain health seems to be mediated by the upregulation of some neurotrophic factors such as brain-derived neurotrophic factor (BDNF), which appears to be highly concentrated in the hippocampus. Additionally, it has an important role in the synaptic plasticity, exerting its effect on synaptic structure and function, neurogenesis, neural survival and disease resistance [13,21,27]. 

*Limitations.* A clear limitation of the current study was the number of patients that may indicate that the patients included in this study were potentially a specific sub-group of patients with MCI and positive biomarkers for AD and patients with AD. Also, the large number of variables probably were not proportioned for the number of subjects, limiting the interpretation of the results. 

*Practical implications and conclusions.* The statistical approach that we applied identified an association between exercise capacity and global cognitive and physical function in MCI and AD. The importance of this result is in the applicability of the assessment methods that are easy to implement in a standard clinical setting. Indeed, it requires a 6MWT, which can be performed in any quiet place, and a submaximal VO_2_max test. Concluding, this model could have important uses, such as targeting individuals for treatment as early as possible and preventative strategies in high-risk population groups. This result offers important information that might help in developing new and more effective exercise-based therapeutic strategies to counteract neurodegenerative processes. 

## Figures and Tables

**Table 1:** Demographic and clinical characteristic of the study participants.

Characteristic	M ± SD
MCI, *n* (%)	12 (28.6)
AD, *n* (%)	30 (71.4)
Female, *n* (%)	27 (64.3)
Age, years	78.7 ± 5.7
Weight, kg	72.5 ± 14.5
Height, m	1.64 ± 0.2
IPAQ (METs·min·week^−1^)	3837 ± 589
**Clinical Characteristics**	
Time since diagnoses, years	4.5 ± 2.5
MMSE, (0–30)	24 ± 2.9
CDR, (0–3)	1.25
FAB, (0–18)	9.5 ± 3
IADL, (0–100%)	61.7 ± 32.5
**Health-Related Markers**	
Sys, mmHg	130.4 ± 6.2
Dia, mmHg	87.8 ± 5.2
HCT, L/L	0.42 ± 0.04
Hb, g/L	13.6 ± 1.4
Glicemia, mg/dL	100.5 ± 26.2
HDL, mg/dL	61.9 ± 23.7
LDL, mg/dL	110 ± 23
Trigl, mg/dL	102.7 ± 29.4
Cortisol, nmol/L	14.5 ± 3.4
**Pharmacological Treatment, *n* (%)**	
Cholinesterase inhibitors	14(33.3)
Antipsychotics	4(9.5)
Antidepressants	7(16.6)
Benzodiazepines	2(4.8)
**Comorbidities, *n* (%)**	
Cardiovascular disease	9(21.4)
Diabetes	3(7.1)
Arthrosis	5(11.9)

Note. MCI, Mild Cognitive Impairment; AD, Alzheimer’s Disease; IPAQ, international physical activity questionnaire; MMSE, Mini Mental State Examination; CDR, Clinical Dementia Rating Scale; FAB, Frontal Assessment Battery; IADL, independent activities of daily living; Sys and Dia, systolic and diastolic blood pressure; HCT, hematocrit; Hb, hemoglobin; HDL, high-density lipoprotein; LDL low-density lipoprotein; Trigl, triglycerides. Values are expressed as mean ± standard deviation (or percentage in parentheses).

**Table 2:** Assessed variables of patients with MCI with positive markers for AD and patients with AD.

Markers	M ± SD
**Bain volume**	
Non normalized, mL	928 ± 119
Normalized for skull size, mL	1354 ± 75
**Physical and bioenergetic variables**	
6MWT, m	365 ± 87
VO_2_max, mL·kg·min^−1^	25.8 ± 5.9
**Vascular function and inflammatory markers**	
VEGF, pg/mL	31.9 ± 10.5
TNF-a, pg/mL	0.714 ± 0.015
IL-15, pg/mL	29.1 ± 23.1
FMD, %	7.8 ± 2.6
FMD/Shear	0.1015 ± 0.149
Shear Rate	175,618 ± 111,627
**Lymphocytes markers and receptors**	
β-NGF, pg/mL	103.9 ± 51.8
TrkA Mono, %	93.7 ± 7.3
p75 Mono, %	81.4 ± 9.4
MFI TrkA	153.6 ± 101.7
MFI p75	23.9 ± 10.7
IL-6, %	6.5 ± 2.1
IL-10, %	4.4 ± 2.4

Note. MCI, Mild Cognitive Impairment; AD, Alzheimer’s Disease; MMSE, Mini Mental State Examination; PPT, physical performance test; 6MWT, 6-min walking test; VO_2_max, maximal oxygen uptake; VEGF, vascular endothelial growth factor; TNF-α, tumor necrosis factor-α; IL-15, interleukin-15; β-NGF, β-nerve growth factor; TrkA, tropomyosin-related kinase receptor; p75, p75receptor; Mono, monocytes; PBMC, peripheral blood mononuclear cells; MFI: mean fluorescence level. Values are expressed as mean ± standard deviation.

**Table 3:** Pearson correlation coefficients between MMSE, PPT and all measured variables.

Variables	MMSE		PPT	
	*p*	R	*p*	R
MMSE	-----	-----	-----	-----
PPT	-----	-----	-----	-----
Age	0.331	0.198	0.298	0.201
Gender	0.276	0.159	0.300	0.299
Weight	0.199	0.174	0.250	0.183
Non-correted Brain Volume	0.194	0.023	0.168	0.037
Corrected Brain Volume	0.202	0.129	0.198	0.248
IADL	<0.001	0.664	<0.001	0.725
6MWT	<0.001	0.627	<0.001	0.749
VO_2_max	<0.001	0.662	<0.001	0.490
VEGF	0.789	0.178	0.876	0.112
TNF-a	0.179	0.292	0.098	0.209
IL-15	0.173	0.341	0.181	0.139
FMD%	0.069	0.298	0.129	0.298
FMD/Shear	0.321	0.210	0.183	0.372
Shear Rate	0.199	0.203	0.193	0.389
β-NGF	0.004	0.453	0.015	0.398
TrkA Mono	0.062	0.302	0.752	0.412
p75 Mono	0.113	0.267	0.018	0.387
MFI TrkA	0.158	0.307	0.083	0.288
MFI p75	0.020	0.372	<0.001	0.497
IL-6	0.210	0.111	0.191	0.234
IL-10	0.157	0.320	0.016	0.499

Note. MMSE, mini mental state examination; PPT, physical performance test; 6MWT, 6-min walking test; VO_2_max, maximal oxygen uptake; VEGF, vascular endothelial growth factor; TNF-α, tumor necrosis factor-α; FMD, flow-mediated dilation; β-NGF, β-nerve growth factor; TrkA, tropomyosin-related kinase receptor; p75, p75 receptor, Mono, monocytes; PBMC, peripheral blood mononuclear cells; MFI, mean fluorescence level. ns, non statistically significant

**Table 4:** MMSE multiple regression model.

Variable	Coeff.	S Coeff.	SE	*P*
Costant	13.384		2.864	
6MWT	0.00599	0.131	0.00863	0.029
VO_2_max	0.235	0.341	0.130	0.087
MMSE = 11.384 + (0.00599 × 6MWT) + (0.235 × VO_2_max)				

**Note.** Coeff., coefficient; S Coeff., standard coefficient; SE, standard error, *P*, *p*-value; 6MWT, 6-min walking test, VO_2_max, maximal oxygen uptake

**Table 5:** PPT multiple regression model.

Variable	Coeff.	S Coeff.	SE	*P*
Constant	1.848		4.502	
6MWT	0.0264	0.698	0.00491	<0.001
VO_2_max	19.693	0.251	10.197	0.079
PPT = 1.848 + (0.0264 × 6MWT) + (19.693 × VO_2_max)				

**Note.** Coeff., coefficient; S Coeff., standard coefficient; SE, standard error, *P*, *p*-value; 6MWT, 6-min walking test; VO_2_max, maximal oxygen uptake.
